# Oocyte-granulosa-theca cell interactions during preantral follicular development

**DOI:** 10.1186/1757-2215-2-9

**Published:** 2009-07-09

**Authors:** Makoto Orisaka, Kimihisa Tajima, Benjamin K Tsang, Fumikazu Kotsuji

**Affiliations:** 1 Department of Obstetrics & Gynecology, University of Fukui, Matsuoka, Fukui, 910-1193, Japan; 2 Reproductive Biology Unit and Division of Reproductive Medicine, Department of Obstetrics, University of Ottawa, Ontario, Canada; 3 Gynaecology and Cellular & Molecular Medicine, University of Ottawa, Ontario, Canada; 4 Chronic Disease Program, Ottawa Hospital Research Institute, The Ottawa Hospital (Civic Campus), Ottawa, Ontario, K1Y 4E9, Canada; 5 World Class University Major in Biomodulation, Department of Agricultural Biotechnology, College of Agriculture and Life Sciences, Seoul National University, South Seoul 151-921, South Korea

## Abstract

The preantral-early antral follicle transition is the penultimate stage of follicular development in terms of gonadotropin dependence and follicle destiny (growth versus atresia). Follicular growth during this period is tightly regulated by oocyte-granulosa-theca cell interactions. Formation of the theca cell layer is a key event that occurs during this transitional stage. Granulosal factor(s) stimulates the recruitment of theca cells from cortical stromal cells, while oocyte-derived growth differentiation factor-9 (GDF-9) is involved in the differentiation of theca cells during this early stage of follicular development. The preantral to early antral transition is most susceptible to follicular atresia. GDF-9 promotes follicular survival and growth during transition from preantral stage to early antral stage by suppressing granulosa cell apoptosis and follicular atresia. GDF-9 also enhances preantral follicle growth by up-regulating theca cell androgen production. Thecal factor(s) promotes granulosa cell proliferation and suppress granulosa cell apoptosis. Understanding the intraovarian mechanisms in the regulation of follicular growth and atresia during this stage may be of clinical significance in the selection of the best quality germ cells for assisted reproduction. In addition, since certain ovarian dysfunctions, such as polycystic ovarian syndrome and gonadotropin poor-responsiveness, are consequences of dysregulated follicle growth at this transitional stage, understanding the molecular and cellular mechanisms in the control of follicular development during the preantral-early antral transition may provide important insight into the pathophysiology and rational treatment of these conditions.

## Introduction

**Figure 1:**
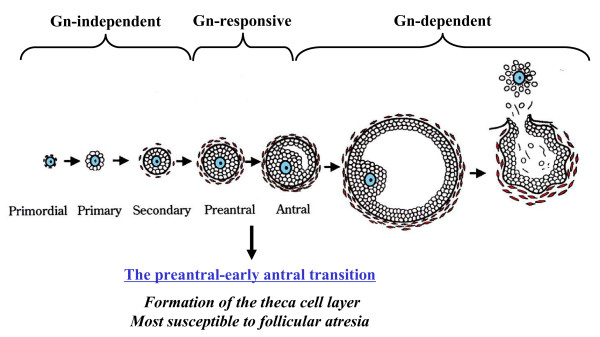
**The transition of the follicle from the preantral to early antral stage is the "penultimate" stage of development in terms of gonadotropin (Gn) dependence and follicle destiny (growth versus atresia)**.

The ovarian follicle, consisting of an oocyte surrounded by granulosa and theca cells, represents the basic functional unit of the ovary. Follicular growth can be classified into three phases according to their developmental stage and gonadotropin dependence [1-3] (Fig. 1): (1) follicular growth through primordial, primary, and secondary stages (gonadotropin-independent phase), (2) transition from preantral to early antral stage (gonadotropin-responsive phase), and (3) continual growth beyond the early antral stage (gonadotropin-dependent phase), which includes follicle recruitment, selection, and ovulation [4]. In the second (gonadotropin-responsive) phase, growth of the follicles is primarily controlled by intraovarian regulators (e.g., growth factors, cytokines, and gonadal steroids) and does not require gonadotropins for growth [5,6], although it is also stimulated by the presence of FSH [1,7,8].

The transition of the follicle from the preantral to early antral stage is the "penultimate" stage of development in terms of gonadotropin dependence and follicle destiny (growth versus atresia) [9] (Fig. 1). Follicles selected for further development are thought to receive precise gonadotropic and intra-ovarian regulatory signals for survival, whereas follicular atresia is a consequence of inadequate growth support [10]. As the preantral-early antral transition is most susceptible to follicular atresia [1,11], understanding the intraovarian mechanisms in the regulation of follicular growth and atresia during this stage may be of clinical significance in providing germ cells for assisted reproduction. Since ovarian dysfunctions, such as polycystic ovarian syndrome (PCOS) and gonadotropin poor-responsiveness, are consequences of this transitional stage-specific dysregulated follicle growth [3], understanding the molecular and cellular mechanisms in the control of follicular development during the preantral-early antral transition may provide important insight into the pathophysiology of these conditions. This review will focus on recent progress that has been made in understanding the importance of intraovarian cell-cell interactions during follicular development from preantral to early antral stage.

### Formation of the theca cell layer

The role of theca cells in follicular function has received less attention compared with intensive investigation into the role of granulosa cells [12]. Nevertheless, the appearance of a theca cell layer at the preantral stage is an important physiological event for early follicular development, as evidenced by: 1) the concurrence of the organization of the theca cell layer and the increased follicular growth and steroidogenic response to gonadotropins [13,14]; 2) increased structural support by the theca cell layer and blood supply containing ovarian regulators for the developing follicle [15,16]; and 3) increased thecal aromatizable androgen production for granulosa cell estrogen biosynthesis and enhanced early follicular growth by androgenic products of the theca cell [17-21].

#### Granulosa-stromal (pretheca) cell interaction

The origin of theca cells has been a long-standing research interest and whether the cortical or medullary stromal cells are thecal stem cells remains an unanswered question. Our recent studies with a bovine co-culture model [22,23] indicates that cortical but not medullary stromal cells are actively transformed into theca cells by the presence of granulosa cells, a process associated with increased LH receptor (LHR) mRNA expression and androstenedione production [24]. These findings suggest that granulosa cells play a decisive role in the differentiation of cortical stromal cells into LH-responsive steroidogenically active theca cells by the secretion and action of a soluble factor(s). In support of this theory, an array of paracrine factors from granulosa cells governing theca cell differentiation have been reported in humans [25]. Huang et al. reported that the combination of two granulosa cell-produced peptides, *i.e. *insulin-like growth factor-I (IGF-I) and kit ligand (KL), increased gene expression for androgenic factors and androgen production in rat theca-interstitial cells [26]. Using the bovine ovarian organ culture model, Parrott and Skinner also reported that KL stimulated ovarian stromal cell proliferation, whereas it had no effect on androgen production [27]. Theca cells maintain epithelial-like appearance and androgenic capacity when co-cultured with granulosa cells, but become fibroblastic and produce less androgen when cultured alone [22], suggesting that the presence of granulosal factor(s) is indispensable for theca cells to sustain their morphology and function.

#### Oocyte-theca cell interaction

Oocyte-somatic cell interaction plays a critical role in folliculogenesis, including activation of resting follicles, early growth, and terminal differentiation [28-31]. Growth differentiation factor-9 (GDF-9) is an oocyte-derived factor and a member of the TGF-β superfamily, which includes TGF-β, activin, and bone morphogenetic proteins (BMPs) [32,33]. Ovaries from GDF-9 null mice exhibit a developmental block at the primary follicle stage, which is characterized by failed theca cell layer formation in early follicles [34]. These observations raise the possibility that GDF-9 also stimulates theca cell recruitment, proliferation and differentiation, and induces the formation of theca cell layer during this early stage of the follicular development. Nevertheless, GDF-9 is believed to be more important for the differentiation than the recruitment of theca cells, since the double-mutant (GDF-9 and inhibin α) mouse exhibits preantral follicles with theca cells having typical morphology but undetectable selective thecal markers, CYP17A1 and LH receptor [35]. GDF-9 treatment increases androgen production in cultured rat theca-interstitial cells [36] and promotes murine ovarian expression of the specific theca cell marker CYP17A1 [34]. GDF-9 increases theca cell number and DNA synthesis in theca cells of small bovine follicles [37]. We recently indicated that GDF-9 augments androgen production and CYP17A1 mRNA expression in rat preantral follicles, whereas down-regulation of GDF-9 by intra-oocyte injection of GDF-9 Morpholino antisense oligos suppressed these responses, indicating that GDF-9 is important in theca cell differentiation during preantral-early antral transition [38].

### Follicular growth and atresia during the preantral-early antral transition

In mammals, a single or small number of germ cell(s) will ovulate during an ovarian cycle, whereas most follicles undergo atresia by follicle cell apoptosis [1,3,15], a selection process that ensures the release of only the healthiest and most viable oocytes [39,40]. Cell apoptosis is triggered by activation of a series of cysteine aspartate-specific proteases (caspases), which include initiator caspases (*e.g. *caspase-8 and -9) and effector caspases (*e.g. *caspase-3). Although apoptosis can occur at all stages of follicular development, the early antral follicles (diameter: 200–400 μm in rats, 2–5 mm in human) are most susceptible to atreatogenic signals [1,11,41]. In contrast, minimal atresia or granulosa cell apoptosis is evident in preantral and the smallest antral follicles (diameter: <200 μm in rats, <2 mm in human) [15,42]. Accordingly, the preantral to early antral transition is the penultimate stage of development in terms of gonadotropin dependence and follicle destiny (survival/growth vs. atresia) [9]. Follicular growth and atresia during this transitional stage is mainly regulated by intrafollicular regulators, such as growth factors, cytokines, and steroids.

#### Oocyte-granulosa cell interaction

Deletion of GDF-9 in the oocyte results in decreased granulosa cell proliferation, abnormal oocyte growth, and failure of follicles to develop past the primary stage [43], demonstrating the importance of this growth factor in early follicular development. GDF-9 stimulates rat granulosa cell proliferation, cumulus cell expansion, and preantral follicle growth *in vitro *[44]. We have recently demonstrated that GDF-9 down-regulation attenuates both basal and FSH-induced follicular growth *in vitro*, while the addition of recombinant GDF-9 enhances basal and FSH-induced follicular growth in rat [9]. In addition, down-regulation of GDF-9 content increases caspase-3 activation and granulosa cell apoptosis [9]. GDF-9 was sufficient to suppress ceramide-induced apoptosis in primary granulosa cells from early antral, but not large/preovulatory follicles [9], suggesting that GDF-9 is an important granulosa cell survival factor during the preantral to early antral transition, but may play a lesser role in follicle survival past antrum formation. GDF-9 also promotes development and survival of human early follicles in organ culture [45]. There may be considerable crosstalk between GDF-9 and FSH during the preantral-early antral transition, as GDF-9 is required to maintain FSH receptor expression in the preantral follicles [9], and GDF-9 receptors (BMPRII and ALK-5) are up-regulated by co-treatment of estrogen and FSH [46]. Although bone morphogenic protein-15 (BMP-15), another oocyte-specific member of the TGF-β superfamily, is also an important regulator of ovarian function [33], whether its action in granulosa cells is anti-apoptotic during this transitional stage and important in protecting the preantral follicles from undergoing atresia remains unknown.

#### Oocyte-theca cell interaction

Ovarian androgens are produced by theca cells, and act via receptors (AR) localized to granulosa cells, stromal cells, and oocytes [47]. Inactivation of AR in female mice results in premature ovarian failure, indicating that normal folliculogenesis requires AR-mediated androgen action [48,49]. AR expression is highest in granulosa cells of rat small preantral and early antral follicles [50], raising the possibility that androgens are important paracrine regulators of follicular growth during preantral to early antral transition. Although androgens have long been implicated as an inhibitor of antral follicular development [51,52], recent evidence suggests that the effect of androgens on follicular growth is dependent on the stage of follicular development and that androgens also have a growth promoting role in early folliculogenesis. Administration of androgens to adult rhesus monkeys significantly increased the number of preantral and small antral follicles as well as granulosa and theca cell proliferation [17]. *In vitro *studies have shown that androgens stimulate preantral follicle growth and granulosa cell mitosis in mice [19], the transition of primary follicles to secondary follicles in cattle [53], and follicular survival in human [54].

We have recently shown that oocyte-derived GDF-9 enhances rat preantral follicle growth, and augments androgen production and CYP17A1 mRNA expression in the preantral follicles, whereas down-regulation of GDF-9 suppressed these responses [38]. The specific AR antagonist flutamide suppressed GDF-9-induced preantral follicle growth *in vitro *[38]. The non-aromatizable androgen DHT, but not estradiol, rescued the follicular growth arrest by GDF-9 down-regulation [38], indicating that androgens exert a direct stimulatory action on the follicular development, especially during the preantral-early antral stage transition. These results suggest that GDF-9 promotes rat preantral follicle growth by up-regulating theca cell androgen production.

#### Theca-granulosa cell interaction

Evidence indicates that steroidal and nonsteroidal factors produced by granulosa and theca cells influence proliferation and differentiation of both cell types on opposite sides of a basement membrane during folliculogenesis [1,7,15,55-58]. LH receptors are found exclusively on theca cells and FSH receptors exclusively on granulosa cells during preantral follicle development. LH stimulates theca cell androgen and growth factor production, while FSH induces aromatase expression and increases the conversion of theca cell androgen to estrogen (two-cell two-gonadotropin theory [59]). Although growth beyond the small antral follicle is characterized by increased aromatase activity and follicular estrogen production, the aromatase activity before the small antral stage is limited [47], suggesting that androgen plays a more important role than estrogen during the preantral to early antral transition. It has been reported that androgens stimulate preantral follicle growth and granulosa cell mitosis [17,19]. Androgens also enhance FSH action in the follicles by increasing FSH receptor expression, FSH-induced granulosa cell aromatase activity and proliferation, and follicular growth [18,47,60,61]. Although we have shown that GDF-9 is required for the expression of FSH receptor in rat preantral follicles [9], whether this response is modulated through thecal androgen actions awaits further investigation. Although it has been demonstrated that LH stimulates follicular maturation [62,63] and induces follicular atresia [64], recent studies suggest that LH is also a stimulant for early stages of follicular growth [12,65,66].

Our previous studies suggest that theca cell-derived soluble growth factors promote granulosa cell proliferation [22] and suppress granulosa cell apoptosis [12] in early, but not large, antral follicles. Although the nature of the theca-granulosa cell interaction remains to be determined, recent studies also suggest the importance of this interaction in the regulation of apoptosis of granulosa cells. Epidermal growth factor (EGF), TGF-α, keratinocyte growth factor (KGF), hepatocyte growth factor (HGF), and BMP-7 appear to be potential physiological inhibitors of apoptotic cell death in the ovary [27,67-71]. These growth factors produced by theca cells might be one of the factors that decreased the incidence of apoptosis in granulosa cells during the preantral/early antral transition.

## Conclusion

**Figure 2:**
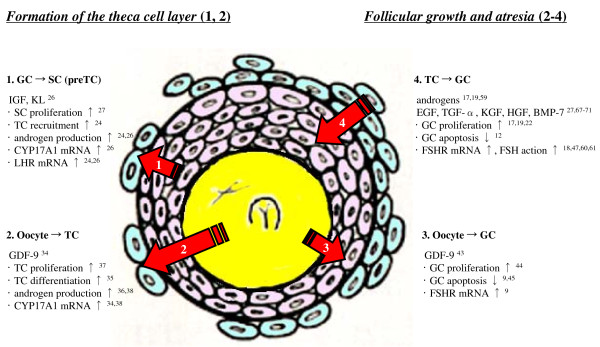
**Follicular growth during the preantral/early antral transition is tightly regulated by intra-ovarian oocyte-granulosa-theca cell interactions**.

The preantral to early antral transition is the penultimate stage of follicular development in terms of gonadotropin dependence and follicle destiny (growth versus atresia). Follicular growth during the preantral-early antral transition is tightly regulated by intra-ovarian oocyte-granulosa-theca cell interactions (Fig. 2). Formation of the theca cell layer is a key event that occurs during this transitional stage. Granulosal factor(s) appears to stimulate the recruitment of theca cells from cortical stromal cells, while oocyte-derived GDF-9 is involved in the differentiation of theca cells during this early stage of follicular development. The preantral to early antral transition is most susceptible to atreatogenic factors. GDF-9 also promotes follicular survival and growth during the preantral to early antral transition by suppressing granulosa cell apoptosis and follicular atresia. GDF-9 enhances preantral follicle growth by up-regulating theca cell androgen production. Thecal factor(s) also promote granulosa cell proliferation and suppress granulosa cell apoptosis. The challenge ahead is not only understand the precise nature of these interactions, but also how they interact in the regulation of follicle destiny, and how dysregulation in these interactions may lead to ovarian pathology such as PCOS and gonadotropin poor-responsiveness. In addition, identification of the factor(s) that promote follicle growth from the preantral stage to small antral stage may provide important information for the identification of intra-follicular biomarkers for the selection of healthy oocytes and embryos in assisted reproduction.

## Abbreviations

GDF-9: growth differentiation factor-9; PCOS: polycystic ovarian syndrome; LHR: LH receptor; IGF-I: insulin-like growth factor-I; KL: kit ligand; TGF-β: transforming growth factor-β; CYP17A1: 17α-hydroxylase/17,20 lyase; caspases: cysteine aspartate-specific proteases; BMP-15: Bone morphogenetic protein-15; ALK-5: activin-like receptor kinase-5; BMPRII: BMP receptor type II; AR: androgen receptor; DHT: 5α-dihydrotestosterone; EGF: epidermal growth factor; KGF: keratinocyte growth factor: HGF: hepatocyte growth factor; GC: granulosa cell; TC: theca cell; SC: stromal cell; FSHR: FSH receptor.

## Competing interests

The authors declare that they have no competing interests.

## Authors' contributions

MO and KT participated in drafting the full manuscript and creating figures. BKT and FK participated in substantial contribution to conception and revising it critically for important intellectual content. All authors read and approved the final manuscript.
